# The ENJOY Seniors Exercise Park IMP-ACT project: IMProving older people’s health through physical ACTivity: a hybrid II implementation design study protocol

**DOI:** 10.1186/s13690-024-01262-z

**Published:** 2024-03-26

**Authors:** Pazit Levinger, Marcia Fearn, Bronwyn Dreher, Adrian Bauman, Natasha K. Brusco, Andrew Gilbert, Sze-Ee Soh, Elissa Burton, Lisa James, Keith D. Hill

**Affiliations:** 1 National Ageing Research Institute, Royal Melbourne Hospital, PO Box 2127, Melbourne, VIC 3050 Australia; 2 Institute for Health and Sport, Victoria University, Melbourne, Australia; 3 Rehabilitation, Ageing and Independent Living Research Centre, Monash University, Melbourne, Australia; 4 Faculty of Medicine, Dentistry and Health Sciences, University of Melbourne, Melbourne, Australia; 5 School of Public Health, and the Charles Perkins Centre, University of Sydney, Sydney, Australia; 6 Department of Social Inquiry, La Trobe University, Melbourne, Australia; 7 School of Primary and Allied Health Care, Monash University, Melbourne, Australia; 8 Curtin School of Allied Health, Faculty of Health Sciences, Curtin University, Perth, Australia; 9 enAble Institute, Faculty of Health Sciences, Curtin University, Perth, Australia

**Keywords:** Seniors Exercise Park, Physical activity, Exercise, Built environment, Older people

## Abstract

**Introduction:**

The health benefits of physical activity are well established; however, most older people are not sufficiently physically active. Despite the availability of various physical activity interventions and programs, implementation of effective prevention strategies to reduce older people’s physical inactivity are lacking. The ENJOY IMP-ACT project is an implementation research project, based on a previous evidence-based physical and social activity program utilising specialised outdoor exercise equipment (the Seniors Exercise Park) for older people. The ENJOY IMP-ACT aims to increase participation in physical activity to improve health outcomes for older people in Victoria, Australia.

**Method:**

The ENJOY IMP-ACT is a hybrid II implementation-effectiveness pre-post mixed method study design. Five local governments (6 public sites/parks) will undergo a 3-month control period followed by 9-months implementation intervention (TERM framework intervention: Training, Engagement, Resources development, Marketing and promotion), and a maintenance phase (3 months). Various methodologies will be employed throughout the project at each site and will include direct observations of park users, intercept surveys with park users, online access monitor platform (using an online app), interviews with stakeholders and exercise program leaders, a process evaluation of physical activity programs, a social return-on-investment analysis, and other related activities.

**Discussion:**

Through the implementation framework design, the ENJOY IMP-ACT is uniquely placed to translate an evidenced-based physical and social activity program into real world settings and increase physical activity among older people. If successful, this program will inform scale up across Australia with the goal of improving the health and wellbeing of older people.

**Trial registration:**

This registration trial is prospectively registered with the Australian New Zealand Clinical Trials Registry. Trial number ACTRN12622001256763. Date registered 20/09/2022.

## Background

Higher sedentary behaviour and physical inactivity are strongly associated with all-cause mortality, increased chronic disease, age-related multi-morbidity, functional dependence, and poorer mental health outcomes in older age [1–3]. Physical inactivity costs health-care systems over $53.8 billion (INT$) annually worldwide [4]. An adequate level of physical activity is known to reduce the risk of health problems in older people [5–7] and can also prevent or ameliorate age-related multi-morbidity [3]. Yet only 25% of older Australians meet the recommended physical activity guidelines [8]. Green space, parks and outdoor leisure are essential for our mental and physical health, as they play an important role in increasing engagement in physical activity [9, 10]. Furthermore, the health benefits of outdoor spaces and parks for older people in particular are also well known [11].

Despite evidence demonstrating the importance of physical activity, the participation of older populations in physical activity programs is low [12]. Effectively translating and sustaining programs in real world settings is a complicated and lengthy process [13]. Few evidence-based physical activity interventions have been effectively implemented into real-world settings [14]. Hence a priority is to identify programs that can be scaled up, understand factors affecting implementation, and establish a framework to guide successful research translation and program sustainability [13]. We previously developed the Seniors Exercise Park program, an innovative outdoors exercise program using specialised outdoor exercise equipment, designed to promote community health and wellbeing through the provision of a unique exercise and social support program. We have demonstrated the effectiveness of the Seniors Exercise Park program in a randomised controlled trial [15, 16] and a community translation research project (ENJOY project) [17]. The ENJOY project provided further evidence that our program promotes sustained engagement of older people in physical activity, in addition to improving physical function, wellbeing, quality of life, and reduced risk of falls [17, 18]. The social aspects and the perceived health benefits (e.g., better health, improved balance, strength, mobility) were key facilitators of ongoing participation [19]. Our work with communities and local governments highlights the potential of this innovative approach to improve older people’s lives through changes in the built environment [20]. Participation in the program is also likely to reduce social and health-care costs [21]. Through our ongoing work with local governments, we have developed a framework to build capacity and knowledge in the community for sustainable impact with the aim of increasing older people’s park visitation and physical activity [22]. Consequently, the next stage is to scale up this innovative approach across additional local governments (municipalities) in Victoria, Australia (including regional areas) and evaluate the process of translating this project across the community and assessing its impact at a larger scale.

The aims of the present study, the ENJOY Seniors Exercise Park IMP-ACT project (ENJOY IMP-ACT: IMProving older people’s health through physical ACTivity), are to evaluate: (1) the effectiveness of the implementation framework on increasing uptake and usage of the Seniors Exercise Park across six local areas and (2) the impact of the ENJOY Seniors Exercise Park on older people’s physical activity and wellbeing, and (3) the social return-on-investment (SROI).

## Methodology

### Study objectives

- Aim 1 – to evaluate the effectiveness of the implementation framework on increasing uptake and usage of the Seniors Exercise Park across six sites within five local government regions. Specifically, we will evaluate:
-
  - the number of older people engaged in physical activity utilising the Seniors Exercise Parks
  - the types of usage and uptake (e.g., organised programs, independent usage)
  - the contexts and mechanisms (barriers/facilitators) influencing implementation.
- Aim 2 – to improve physical activity and wellbeing of older people using the equipment. Specifically, we will evaluate:
  - the physical activity and wellbeing outcomes of older people utilising the equipment
  - older people’s usage characteristics of the equipment (e.g., frequency, duration).
- Aim 3 – to evaluate the social return-on-investment when scaling the Seniors Exercise Park across multiple local government areas.

### Study design

All procedures involved in this trial will be conducted in compliance with the National Statement on Ethical Conduct in Human Research and the Australian Code for the Responsible Conduct of Research. The study was approved by the Monash University Human Research Ethics Committee, Melbourne Australia (Project ID: 35502). The study was designed according to the Strengthening The Reporting of Observational studies in Epidemiology (STROBE) statement [23]. STROBE check list is provided as an additional file.

**Fig. 1:**
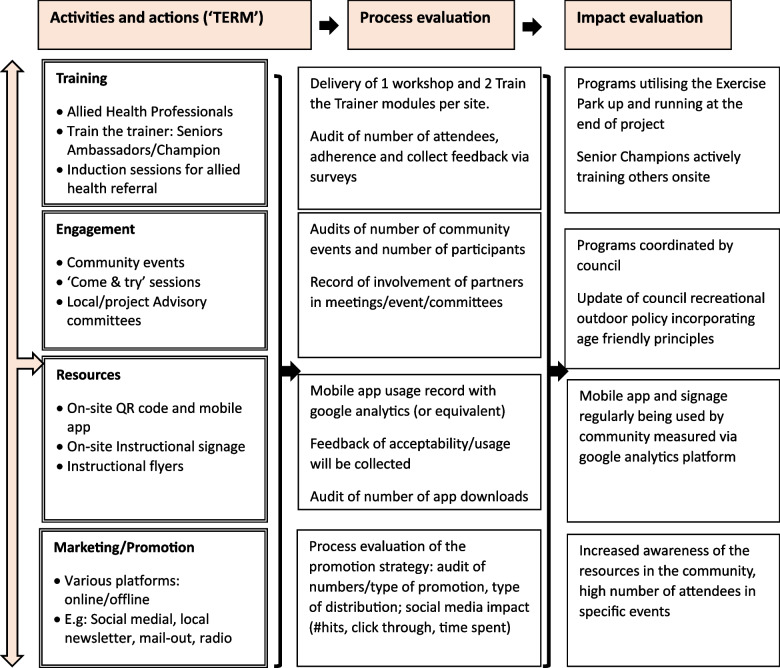
The ENJOY IMP-ACT implementation activities, process and impact evaluation using the RE-AIM Framework and study-specific adopted logic model

**Fig. 2:**
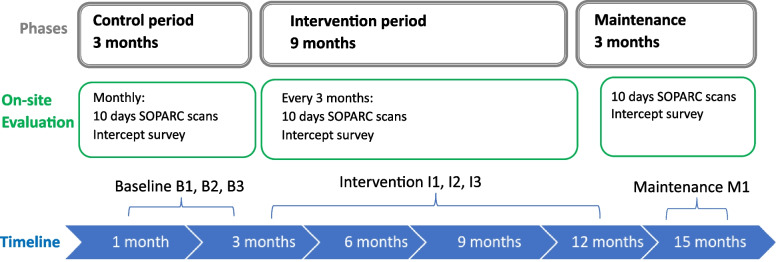
Project’s phases at each site

The ENJOY IMP-ACT study is an implementation research project employing a hybrid II implementation-effectiveness pre-post mixed method design [24]. We will implement and evaluate the implementation intervention, process and impact using the Reach, Effectiveness, Adoption, Implementation, and Maintenance (RE-AIM) Framework [25] and our adopted logic model (see Fig. 1). Six sites in Victoria, including two regional sites, will participate in the study with a pragmatic staggered commencement. Each site will have a 3-month control period followed by 9-months implementation intervention (TERM framework intervention: Training, Engagement, Resources development, Marketing and promotion), and a maintenance phase (3 months) (Fig. 2). The pre-post design is a pragmatic real-world design where all local governments will receive the implementation intervention framework. A control period will act as the waiting list ‘control arm’.

To address the study aims, multiple methodologies will be employed, including: direct observations of park users, intercept surveys with park users, online access monitoring platform (using an online mobile application), interviews/surveys with stakeholders and exercise program leaders, and process evaluation (review/audits of physical activity programs and other related activities).

#### Study setting and partners

In this study, five councils (six parks/sites) will participate in the project (four in metropolitan Melbourne and one in regional Victoria, Australia), including: City of Whittlesea (metropolitan), Nillumbik Shire Council (metropolitan), Hobsons Bay City Council (metropolitan), Wyndham City Council (metropolitan), and Mitchell Shire Council (two sites in regional Victoria). The proportion of the older population among these five local government ranges between 10.9% to 22% (people 60 years and over) and 7.4% to 17% (people 65 years and over) [26].

The design of the project aims for implementation in staggered stages of commencement in the following order: Barry Rd, Thomastown (City of Whittlesea); Andrew Pocket Park, Eltham (Nillumbik Shire Council); Donald Mclean Reserve, Spotswood (Hobsons Bay City Council); Central Park, Hoppers Crossing (Wyndham City Council); The Elms Reserve, Kilmore; and Chittick Park, Seymour (Mitchell Shire Council). Details of each park location, features and the surrounding areas are provided in Table 1. The locations of the metropolitan parks are spread as follows: 15–20 km north and north-east of the Melbourne Central Business District (CBD) (Thomastown and Eltham), 7 km south-west of Melbourne's CBD (Spotswood) and 24 km south-west of Melbourne's CBD (Hoppers Crossing). The two regional sites are located 65-104 km north of Melbourne (Kilmore and Seymour respectively).

**Table 1:** Parks amenities and features

Amenities /features	Barry Road Community Activity Centre, Thomastown	Park Andrew Pocket Park, Eltham	Donald Mclean Reserve, Spotswood	Central Park Community Centre, Hoppers Crossing	The Elms Reserve, Kilmore	Chittick Park, Seymour
Within Seniors Exercise Park area						
Seniors Exercise Park equipment	Yes	Yes	Yes	Yes	Yes	Yes
Table and seats	2 benches, 1 × picnic table with chairs	2 benches, 1 × picnic table with chairs	1 bench	2 benches, 1 × picnic table with chairs	1 bench, 2 × picnic tables with chairs	2 benches
Shade Sail	Yes	Yes	No	Yes	No	No
Surrounding area adjacent to the Seniors Exercise Park						
Sport play court/field (e.g. oval/basketball/tennis court/netball)	Tennis court	Tennis court, mini-basketball court	Football oval	Pétanque	No	Football ovals
Water fountain	No	Yes	Yes	No	No	Yes
Public Toilet	No	Yes	Yes	No	No	No
BBQ / Picnic area	Yes	Yes	Yes	Yes	Yes	Yes
Seated benches	Yes	Yes	Yes	Yes	Yes	Yes
Roofed shaded area	Over picnic table	Over picnic table	Over picnic table	No	Over picnic table	Over picnic table
Kids playground / play space	1–2	1–2	≥ 2	≥ 2	1–2	≥ 2
Sand pit	No	No	Yes	No	No	Yes
Table and seats	Yes	Yes	Yes	Yes	No	Yes
Other Exercise equipment	No	No	Yes	No	No	No
Walking track	No	Yes	Yes	Yes	Yes	No
Garden beds and landscaping	Yes	Yes	Yes	Yes	No	Yes
Trees	Yes	Yes	Yes	Yes	Yes	Yes
Dog area (off leash/fenced area)	No	No	Yes	No	No	No
Buildings/facility centres	Community centre	Tennis club	Football clubhouse	Community centre	No	Concert band hall
Parking Facilities	Yes	Yes	Yes	Yes	No (only residential street parking)	Yes

**Fig. 3:**
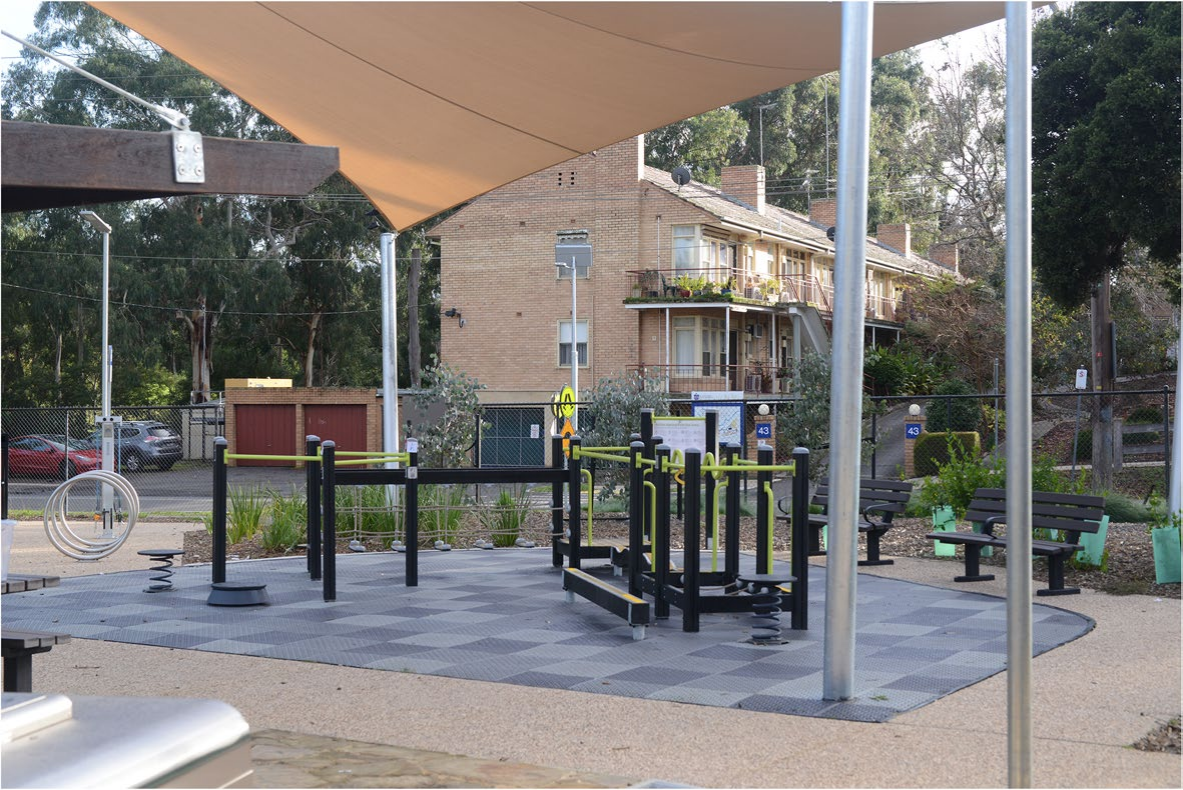
The Seniors Exercise Park in Andrew Pocket Park, Eltham, Melbourne

Each participating council has installed the Seniors Exercise Park and the site is open to the public (Fig. 3). Key staff from each councils’ Positive Ageing/Community Development team (or equivalent) and Open plan/Landscape team (or equivalent) will work closely with the research team to assist with the delivery of the project and to facilitate data collection. A formal agreement (e.g., Memorandum of Understanding (MOU)) will be signed off between the research organisation and each local government prior to commencement of data collection.

Variation in the project’s timeline may occur with potential delays due to weather conditions, future COVID-19 or similar restrictions, local government internal processes, and public holidays. A suitable timeline for the project’s execution and associated data collection will be developed incorporating a contingency plan into the timeline in the study design to account for potential impact of factors outside the researchers’ control (e.g., weather, site refurbishment) which are frequently experienced in natural experiments and pragmatic trials such as this [27, 28].

### Study population

#### Recruitment

Older people visiting the participating parks are potential participants for the intercept surveys and will be recruited at the sites. Information about the study will be provided to residents/visitors via verbal communication as well as via hard copies of the information sheet.

Leaders of delivery programs (seniors group leaders, allied health professionals, exercise instructors), and key stakeholder representatives (council staff, community health/leisure centre managers/coordinators) will be recruited to participate in an interview/survey for the process evaluation.

#### Inclusion criteria

For the observational data collection, all park visitors during park observation periods will be included in the data recording.

For the face-to-face intercept survey at the park during the park observation periods, the following inclusion criteria will be applied: (1) adults aged 60 and over, (2) adults who are able to understand basic English and have conversational English.

For the semi-structured interviews for the contextual factors influencing the implementation, the following inclusion criteria will be applied: (1) key stakeholder representatives—council staff within the division (or equivalent) that are responsible or involved with the Seniors Exercise Park management/coordination/activation; community health/leisure centre managers/coordinators within the participating municipalities; (2) leaders of delivery programs (seniors group leaders, allied health professionals, exercise instructors) who utilise the Seniors Exercise Park as part of their program/service delivery.

#### Exclusion criteria

The following exclusion criteria will be applied for the face-to-face intercept surveys at the park: (1) participants who identify themselves as less than 60 years of age and (2) who are unable to understand basic conversational English.

The following exclusion criteria will be applied for the key stakeholder representatives and leaders of delivery programs interviews: staff that are not directly involved with the management or activation of the Seniors Exercise Park or people who do not deliver/run programs/services using the outdoor equipment.

#### Consent

Consent from park visitors for the observational data collection will not be required; participants remain anonymous, and the behaviour occurs in a public setting where there is no breach of privacy.

Verbal consent will be required to participate in the face-to-face intercept survey. The research staff will explain the study and seek a verbal agreement/consent by the participant prior to commencing the survey. A hard copy of the information sheet will be available from the research staff and will be offered to potential participants should they wish to read and or maintain a copy.

Written consent will be required from stakeholders/leaders of the program to participate in the interviews. Potential participants will receive a written information sheet and a written consent form (hard copy or an electronic copy as a pdf file), via email or in person, and will be required to sign the form (either on the hard copy or electronic signature) and return it to the research team prior to participating (and any data collection). Signed consent forms can be returned to the research team via email (scanned signed copy or e-signature), post, or in person.

#### Procedure

Various methodologies will be employed throughout the project at each site (estimated 15–16 months active process at each site). These will include direct observations of park users, intercept surveys with park users, online access monitor platform (using an online mobile application), interview with stakeholders and exercise program leaders, and process evaluation (review/audits/surveys of physical activity programs and other related activities). The summary of overall methodology is presented in Table 2.

**Table 2:**
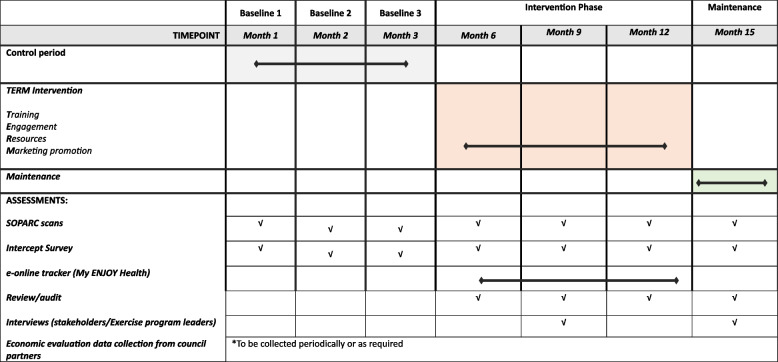
Study schedule per site

##### Intercept surveys

Each site will have a 3-month control period followed by a 9-month implementation intervention period (TERM—Training, Engagement, Resources development, Marketing and promotion), and a maintenance phase (3 months). Evaluation (observation of users/visitors and onsite intercept surveys) will take place each month during the control period (baseline = B), every 3 months during implementation intervention phase (I1, I2, I3), and once during the maintenance phase (M1), see Table 2 and Fig. 2. The control period will include three evaluation points (B1, B2, B3), each occurring over a 10-day period per month (including one weekend). The time series evaluation will enable controlling for variations in equipment access due to weather (seasonal effects). Potential participants (park users in the specified target area) will be approached by trained, clearly identifiable research staff to see if they meet inclusion criteria. They will be provided with a verbal explanation about the study and all ethical considerations and invited to participate in a survey. Interested participants will then be asked to provide a verbal agreement (consent) to participate. Upon verbal consent, a paper survey will be administered by the researcher onsite. If more than one person is in the park during any observation period, the research staff decision of who to approach would be guided by aiming to overall recruit an equal number of men and women. The participants will be eligible to complete only one intercept survey during the baseline and maintenance period. During the intervention period the same participants can be surveyed multiple times.

##### Qualitative semi-structured interviews

For the evaluation of the potential contextual factors (community/organisational level factors) that may influence implementation (barriers/facilitators) of the TERM framework (interviews/surveys), we will reach out to stakeholders and community groups within the participating municipalities using various communication channels: emails, face-to-face engagement at the site or during community events/other forums. Information about the study will be distributed and contact details will be provided for interested individuals.

Eligible participants will be invited to take part in qualitative interviews with a member of the research team. Participants will be given the option of being interviewed face-to-face (where feasible), via video-conferencing, or via telephone. Interviews will be audio-recorded using either handheld recorders or the video-conferencing software and transcribed verbatim by a professional transcription service. Using a semi-structured interview, participants will be asked a predefined series of open questions about the ENJOY IMP-ACT program, and also invited to expand on their answers through follow up questions and offer new topics of discussion.

### Intervention—implementation framework ‘TERM’

**Fig. 4:**
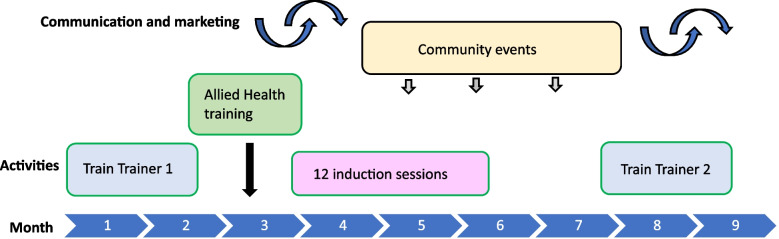
Proposed outlined of the activities to be carried out during the 9 months TERM intervention phase. Variation in the order of activities is expected between sites to accommodate council’s planning and seasonal weather

The implementation intervention is based on our previous piloted implementation framework [20] and includes several elements (‘TERM’, Figs. 1 and 4): **T**raining, **E**ngagement, **R**esources development, **M**arketing and promotion. This includes the core elements of the Interactive Systems Framework (ISF) and the ecologic framework [13, 29]. We identified the ‘TERM’ key elements as important mechanisms in building capacity, knowledge, upskilling and engagement, which thereby facilitate increased usage and uptake of physical activity using the Seniors Exercise Park. Evaluation of the implementation components and associated assessments’ timeline is detailed in Table 3.

**Table 3:** Evaluation of the implementation components using the RE-AIM dimensions and associated assessments’ timeline

	What will be measured (specific aim)	Tool/Measure	Assessment time-point^a^		
RE-AIM dimension			B1-B3	I1-I3	M1
Reach	The number of older people using the equipment (specific to aim 1a)	Modified SOPARC; Seniors Exercise Park observation form; My ENJOY Health usage	✓	✓	✓
	Number of exercise programs (aim 1b)	Audit of programs		✓	✓
Effectiveness	Physical activity level and health of users (aim 2a) Social return on investment (aim 3)	Face to face intercept survey: Self-reported physical activity, Quality of Life, social and health services	✓	✓	✓
Adoption	Number of allied health professional and organisations delivering programs (aim 1b)	Audit		✓	✓
	Number/type of seniors groups utilising the equipment (aim 1b)	Audit, Survey		✓	✓
	Number/type of exercise programs being delivered (aim 1b)	Audit, Survey		✓	✓
Implementation	Details and characteristics of programs being run (aim 1b)	Interview/Survey		✓	✓
	Older people’s usage characteristics of the equipment (e.g., frequency, duration) (2b)	Face to face intercept survey, Mobile app usage		✓	✓
Maintenance	Number of older people/programs/organisations using the equipment (aims 1a, 1b)	Modified SOPARC; Exercise Park observation form; Audit of programs; My ENJOY Health use			✓

^a^ *B1-B3* baseline, *I1-I3* during TERM implementation intervention, every 3 months, *M1* maintenance phase completion

#### ‘TERM’—Training – knowledge transfer

We aim to train allied health professionals and seniors champions, and to work closely with health-care and leisure centre providers in the areas surrounding the participating Seniors Exercise Parks, to support engagement during the trial and continuity beyond the study’s completion.

- *Allied Health Professionals workshops*: A half-day workshop training program will be delivered at each site covering safe use, exercise prescription and program design, incorporating risk management, theoretical and practical sessions.
- *Train the Trainer module (volunteer /champions)* – we will deliver a 5-week training module, 2 days per week (practical and educational components) to older people (based on successful seniors champions training utilised in our research [22]). We will train 6–8 leaders (ENJOY seniors champions) at each site, who will then be able to welcome and train community members on designated days (coordinated by the council). We will deliver 2 modules per site. Upskilling older people as champions can support and empower their peers to maintain participation in physical activity [30]. Using this approach maximises the engagement of the wider community with Seniors Exercise Parks.
- *Induction sessions by qualified research staff (for example, accredited exercise physiologist or physiotherapist)* – 12 sessions will be offered at each site for extra support of park users who may require additional assistance or supervision, and further support for the volunteers (refresher sessions). For example, these sessions may support clients referred from local General Practitioner clinics or community health centres. This will enable transition of high-risk clients from supervised programs to the community with safe induction and familiarisation on the exercise equipment for future independent Seniors Exercise Park usage by these clients.

#### ‘TERM’—Engagement

Engagement with the community, older people, and stakeholders (local governments/health and leisure providers) is important for sustainability as well as effective design of scalable public health interventions [31]. Community engagement has been recognised as a pathway to building trust, encouraging participation, and promoting uptake [32]. We will engage communities through: (1) formal agreement sign-off (MOU with the participating councils), (2) local community events (e.g., open days, Seniors’ Weeks, Community Health Expos), (3) establishment of local advisory groups at each site, and (4) establishment of an overarching project advisory committee (Community of Practice committee). We will document progress in each of these steps as part of the process evaluation. The Community of Practice committee will include local and external stakeholder representatives (e.g., leisure centres, community health centres, council staff from various divisions (e.g., age and disability, community development, open space, leisure and recreation), ENJOY champions, community consumers) and state representatives (e.g., Municipal Association of Victoria, Sports Recreation Victoria, Department of Health).

#### ‘TERM’—Resource development

Our project resources will include information about the benefits of physical activity and safe usage of the equipment. These resources will comprise on-site information (instructional signage with friendly illustrations), traditional hard copy flyers, and an online platform/social media (website, video resources and the online mobile app, My ENJOY Health). We will also develop workshop and training resources (developed with a behaviour change focus), including on-line resources (video materials) and written educational materials (e.g., manuals).

*My ENJOY Health Innovative technology—*we have developed and tested a web mobile application that older people can access on-site via Quick Response (QR) codes fitted on the various exercise stations [22]. The QR codes link to instructions, safety tips, and 48 exercise videos. The existing web app will be upgraded as a native mobile application for iOS and Android (MY ENJOY Health). The platform will be further developed (co-design) to add (1) additional new sites, (2) additional features for engagement, (3) additional data extraction functionality and usage analysis.

#### ‘TERM’—Marketing and promotion

Effective marketing can support and motivate changes in behaviour or practice. Promoting physical activity therefore requires appropriate, relevant and well-resourced marketing to effectively create awareness and knowledge and increase older people’s motivation [33]. We will work closely with the marketing and promotion team within each council to reach older community members, using various targeted platforms: mail out flyers, on-site signage, newsletter stories, local radio stations, social media, and video promotions/photo shoots. As we anticipate variation in the marketing/promotion strategies between councils, we will develop a core strategy that is adaptable based on local structures and resources. This will include a social marketing approach, combining communications with supportive policies, environments and opportunities for physical activity [34].

### Assessments

#### Outcome measures

##### Primary outcomes

The primary impact measures of the implementation framework intervention are: (1) *the number of older people* who engage in physical activity using the Seniors Exercise Park (irrespective of usage mode: e.g., if via group-led programs or independent usage), and (2) *the physical activity level of users*.

Following the hybrid II design, we will evaluate the impact of the intervention and the potential contextual factors (community/organisational level factors) that may influence implementation (barriers/facilitators) of the TERM framework. In addition, we will evaluate older people’s quality of life, wellbeing and self-reported health care utilisation. The RE-AIM model [25] (see measures described in Table 3) and our logic model will guide the project evaluation (Fig. 1).

##### AIM 1a—Primary impact measure of the implementation intervention

The number of older people (users) engaging in physical activity using the Seniors Exercise Park will be evaluated using periodic observation (the System for Observing Play and Recreation in Communities (SOPARC)). The SOPARC is a reliable and feasible instrument for assessing physical activity and associated contextual data in community settings [35]. It is based on momentary time sampling techniques, which systematically and periodically scan individuals and contextual factors within pre-determined target areas in parks. We will use a modified version of the SOPARC, which will record number of visitors, the gender and activity modes/types of people utilising the Seniors Exercise Park [22]. Additional data about the type of usage of the equipment by coding the interaction with the outdoor exercise equipment (i.e., ‘using equipment as intended’ or ‘playing/looking/sitting’ on the equipment) will also be collected [22, 36]. Systematic scans will be conducted over a 10-day period (including weekend) with a total of 14 scans as follows: every 30 min of all park visitors in the study area during early morning (07:30–10:30), mid-day (12:00–13:30) and late afternoon (15:00–17:30) [37]. Evaluation will take place monthly during the control period (Baseline), every 3 months during the implementation intervention phase and once during the three-month maintenance phase. In the event that there are missing scans (e.g. due to days with extreme weather conditions), these scans will be rescheduled to an equivalent day.

##### AIM 2a –Physical activity level of older people users

We have already demonstrated that using the Seniors Exercise Parks improves physical function (objective measures), increases self-rated quality of life and wellbeing, reduces falls risk, and increases physical activity levels [17, 18]. ENJOY IMP-ACT aims to scale up this evidence-based program. We will conduct face-to-face intercept surveys with older people who utilise the equipment using the *self-reported physical activity* questionnaire from the Active Australia Survey (see below for more details) [38]. The survey assesses walking, moderate, and vigorous activity in the previous week, plus providing an indicator of total activity and meeting recommended physical activity guidelines. The Active Australia questions are valid, reliable and recommended for use in Australian population-based research. This will provide measures of time and frequency of physical activities and also identify any changes not likely to be attributable to the program (such as walking) [39], and provide a comparator to nationally representative data.

### Secondary measures

#### Intercept survey with park visitors

##### Users’ physical and health characteristics

Face-to-face intercept surveys (15–20 min) will be conducted at baseline (pre-implementation B1, B2, B3), during the implementation framework (I1, I2, I3), and at the end of the maintenance follow up (M1). Data will be collected during the periodic observation (SOPARC) days. Intercept surveys will provide more in-depth information about Seniors Exercise Park users’ characteristics. The paper-based survey will include a set of questions across various domains similar to previous research [37, 40], as well as validated questionnaires (detailed below). The set of questions will include socio-economic and demographic characteristics of Seniors Exercise Park users (e.g., age group, gender, country of birth, marital status), if they are local residents or visitors, motivation to use the Seniors Exercise Park equipment, how often they visit the park area, social connectedness/engagement with other people at the park area, their general physical activity level and their leisure/recreation activity at the park, and general health and wellbeing. Socio-economic status will be estimated using postcodes to derive the Australian Socio-Economic Indexes for Areas (SEIFA) 2021 Index of Relative Socio-economic Advantage and Disadvantage, where the first and tenth SEIFA decile represents geographical areas with the greatest socioeconomic disadvantage and advantage respectively [41]. Self-reported social and health-care services utilisation (e.g., General Practitioner visits, hospitalisations) for the 3 months prior, as well as leisure activities and occupation details, will also be collected at each time point to provide relevant information for the economic evaluation.

The following validated questionnaires will be used as part of the survey:

- *Health-related quality of life* will be assessed using the EQ-5D-5L [42]. The EQ-5D-5L comprises five dimensions (mobility, self-care, usual activities, pain/discomfort and anxiety/depression), as well as an overall self-rated health status (Visual Analog Scale (VAS) 0–100) where a higher score represents better health. The utility score will be used for the economic evaluation.
- *Self-reported physical activity* will be measured using the Active Australia Survey (Australian Institute of Health and Welfare, 2003 [38]). The Active Australia Survey includes six questions that assess walking, moderate and vigorous activity, in addition to an indicator of total activity. The Active Australia Survey questions were shown to be valid and reliable that can be used in Australian population-based research. The Active Australia Survey questions provide measures of time and frequency spent performing light, moderate and vigorous physical activities as well as an estimate of energy expenditure in metabolic equivalent (MET)-minutes per week.
- *General wellbeing* will be assessed using the five-item World Health Organisation (WHO-5) Wellbeing questionnaire which provides measures of psychological wellbeing and depressive symptoms using 5 simple questions [43, 44]. A percentage score can be calculated using the raw score, which ranges from 0 (representing worst imaginable wellbeing) and 100 (representing best imaginable wellbeing).

##### Type of usage and uptake

We anticipate several ways in which older people will use the equipment: independent usage (e.g., incidental users), organised informal activities (e.g., seniors groups), and structured supervised programs delivered by community centre/leisure organisations. Audits of the number, type of programs (supervised/unsupervised), and program characteristics (duration, frequency, staff profession) will take place at the completion of the intervention via audit and/or survey from the service/program providers.

##### Online access monitor platform – the *My ENJOY Health*

We have developed an innovative online web application (*My ENJOY Health*) to monitor the usage and access of the Seniors Exercise Park by visitors at each site [22]. The online platform will be upgraded with additional features including programs, workouts, specific exercise instructions, videos, and safety tips. QR codes will be placed on the instructional signage and on the exercise equipment itself at each site. Visitors will be able to scan the QR code with their mobile phone at the site or download the native mobile application. The e-monitor tracker platform will collect information on usage of the online platform such as frequency, time, and date of access to the web/and or mobile app. Design and testing of the e-monitor tracker platform will be conducted in the first 3–6 months of the project.

##### Training audit and evaluation

Process evaluation of the training (allied health professional and train the trainer) will include: evaluation of the number/outcomes of workshops/training programs delivered for allied health professionals and seniors champions. A record of the participants will be kept (including their profession and qualification); and a structured evaluation of participants (knowledge gained, subsequent use of the Seniors Exercise Park with clients, and feedback) will be undertaken. Costs will also be collected for the allied health professionals and seniors champions training, as they form part of the investment, in the social return on investment analysis.

##### Contextual factors barriers/facilitators (Aim1c)—community/organisational level factors

To understand community level and organisational factors (e.g., funding, policy, internal structure) that may influence implementation (barriers/facilitators), we will conduct semi-structured interviews with key representatives from partner organisations (local government, and local healthcare /leisure/ recreation providers). In addition, we will conduct semi-structured interviews with leaders of delivery programs (seniors group leaders, allied health professionals, exercise instructors) using the outdoor equipment to understand provider characteristics, and barriers and facilitators experienced throughout their involvement with the Seniors Exercise Parks. The semi-structured interviews with stakeholder representatives and leaders of delivery programs will be conducted one-on-one via telephone, video conferencing or face-to-face. Semi-structured interviews will occur during the intervention and maintenance phases. The interviews will be audio-recorded or video-recorded. Audio-recording will then be transcribed by a professional transcription service.

##### Review and audit of physical activity programs

Information about the type and number of physical activity programs for older people using the Seniors Exercise Park will be provided by the council (from the Positive Ageing team or equivalent) to the research team. There may be different modes of delivery and or programs that will be delivered by the participating partners and/or their respective local health/leisure providers. This information will be collected in the last stage of each site, between 12 and 15 months.

##### Economic evaluation – social-return-on investment (SROI)

The information about the cost investment of participating councils will be collected via an online survey or a bespoke template that will be sent to the community of practice committee members (includes the six local government representatives for each Seniors Exercise Park) frequently at various stages during the trial. The following investment information will be collected and assigned a monetary value: capital costs (purchase, installation and setup); implementation costs (e.g., planning meetings, staff recruitment, marketing, communications, education to Australian Health Professionals and community leaders); running costs (e.g., health professionals, administration team, including the training costs for the allied health professionals and seniors champions) and maintenance costs (e.g., equipment maintenance). To complete the SROI analysis, the investment will be compared to the social return, which will include the monetary value of the Seniors Exercise Park via its impact on participant wellbeing, leisure and employment opportunity, and private and government funded social and health care utilisation (via data collected during the intercept interviews).

### Statistical methods

#### Sample size estimation and justification

##### A target outcome for successful Seniors Exercise Park usage

A NSW study demonstrated only 5.3% (*n* = 6) of older adults (> 60 years) utilise outdoor exercise equipment at a park [36]. In our field work we observed 10 older adults independently using the Seniors Exercise Park in a typical week following installation without any promotion or supported implementation. We anticipate that following the implementation intervention, there will be a 100% increase in the number of independent users (*n* = 20) as well as an increase in the number of older adults using the equipment as part of organised classes (*n* = 30) in a typical week (while accounting for weather impact [45]). Therefore, we would expect there will be approximately 50 equipment users observed over a 10-day period per site. This exceeds the reported fourfold usage increase following park refurbishment reported elsewhere [46]. Power calculations using a Poisson or negative binomial distribution, adjusting for site and weather differences [47] demonstrated that a power of over 0.95 can be achieved to detect a 100% increase in park visitors with this sample size.

##### Sample size calculation for the intercept surveys

Sample size calculations for the intercept surveys as part of aim 2 are based on the expected number of older people who will complete the survey using data from our previous project [22]. We observed 10 older people using the Seniors Exercise Park in a typical week (prior to formal promotion of the newly installed park). We hypothesised that the implementation intervention (TERM) will result in *at least* a twofold increase in the number of older people using equipment at the completion of the intervention phase (I3) compared to baseline (B1,B2,B3), with a 20% attrition rate (proportion of people refusing to be interviewed) during the maintenance phase (M1). Assuming 25% will decline to participate or have previously completed the survey [40], we will aim for a total of 54 intercept surveys (8 at baseline; 34 across I1-I3 and 12 at M1) per site, with an overall sample of 324 interviewees across the six sites. Each data collection period will include an observation period of 10 days (instead of a typical 7-day period as commonly used [35]). Increasing the number of observations days to 10 days will enhance opportunities for the research team to recruit more older visitors to complete the intercept surveys to meet the targeted sample size.

##### Sample size for the semi-structured interviews

For the stakeholder interviews (councils and community health/leisure centre), we aim to interview between 1–2 council staff from each council (5 councils so total of 5–10 interviews), and between 1–2 community health/leisure staff (6 park sites so total of 6–12 interviews). For the leaders of delivery programs (seniors group leaders, allied health professionals, exercise instructors), we aim to interview between 1–2 per site/park, hence a total of 6–12 interviews.

#### Statistical analysis

##### AIM 1—‘TERM’ implementation evaluation and impact

Descriptive statistics will be used to report the overall number of older people using exercise equipment as well as the type of usage and uptake, mobile app access, and survey responses from allied health professionals and seniors leaders. The proportion of missing data will also be reported descriptively, and if more than 10% of data are missing, data will be imputed using multiple imputation techniques. Generalised linear models [47] (with main effects for intervention, site, season and their interaction) will be used to examine the impact of the implementation intervention on the total number of older people (primary outcome) using the equipment with overdispersion handled using a negative binomial distribution. Sensitivity analyses will be undertaken by comparing models with missing data and imputed data.

#### Qualitative analysis

Semi-structured interviews with key stakeholders will be audio recorded, professionally transcribed and analysed thematically using NVivo 12 software (QSR International). Two researchers will follow the six steps of thematic analysis outlined by Braun and Clarke [48]: familiarization, code generation, combining codes into themes, reviewing themes, determining significance of themes, and reporting findings. The researchers will independently code all of the transcripts and then meet to consolidate a shared codebook and seek feedback from the research team, including about any disagreements over codes. This codebook will be applied to coding the transcripts afresh, dividing the transcripts between the researchers. The researchers will then cluster codes into candidate themes. This will be presented to the research team to resolve any disagreements, determine the final themes, and discuss the significance and priority of themes for the study.

##### AIM 2—evaluation of the ENJOY Seniors Exercise Park on physical activity and wellbeing

Summary statistics will be used to describe the demographic, socioeconomic, equipment usage and health-related characteristics of Seniors Exercise Park area users at each time point. Data will also be examined and reported relative to the adult general public, based on the distribution of age, marital status, and country of birth (using Australian Bureau of Statistics census data for each local government area). Linear and logistic regression models (with main effects for site, time-point, season and their interaction) will be used to examine the impact of the implementation intervention on physical activity level (primary outcome) and other health user characteristics. Statistical significance of the interaction term will be used to determine if the outcome varied between sites at each intervention time point (I1-I3) or M1 relative to their baseline difference.

##### AIM 3—economic evaluation – social-return-on investment (SROI)

We will conduct an economic evaluation to determine the SROI for the Seniors Exercise Parks funded by the local governments. Based on pilot work, it is hypothesised that Seniors Exercise Park capital / running costs will be off-set by cost savings of older people who use the Seniors Exercise Parks due to the reduction in private and government funded social and health care costs following participation [49]. This potential reduction in social and health care costs may be associated with health benefits connected with participation in the Seniors Exercise Park. The SROI will be blended with a traditional cost–benefit analysis. The benefits will be tailored to social purposes by including wellbeing, leisure and employment opportunities, in addition to social service and health care utilisation. Costs will include the Seniors Exercise Park capital (purchase, installation and setup), implementation (including the cost of training for allied health professionals and seniors champions), running and maintenance costs. The return/benefit will be modelled based on the difference in reported benefits for older people surveyed prior to, and following, participation in the Seniors Exercise Park; benefits will include the impact on wellbeing, leisure and employment opportunity, and private and government funded social and health care utilisation (to be collected as part of the intercept surveys). Investment costs will take a local government perspective, while the social return will take a broader social perspective. All costs and benefits will be costed at market rate and reported in AUD$2025/26. Where market rates are not available, economic modelling from previous related data sets and the literature will be used.

## Discussion

From 2017 to 2057, Australia’s older population (60 + years) will double to 8.8 million, 22% of the total population [50]. The growth of this demographic and longer life expectancies pose challenges, due to the emergence of many complex health issues associated with an inactive lifestyle, which affect the health and wellbeing of older people. The health benefits of physical activity are well established, including reduced risk of chronic diseases, increased cognitive and functional capacities and improvement in mental health [51, 52]. Physical activity interventions research has proliferated in the past decade but with minimal changes to population physical inactivity. The need for a shift in research to focus on implementation strategies and evidence to support effectiveness of such strategies is warranted [53]. With the ageing population, effective prevention strategies that can be scaled up are essential for populations of older adults.

ENJOY IMP-ACT is based on over 10 years of research work and evidence, supported by the growing popularity of the establishment of age-friendly parks in Australia as a means of creating spaces for older people to engage in physical and social activities in their local communities. Collaboration with local governments and community engagement appear to be an important element in co-creating change in the community [20, 22]. Using an implementation-effectiveness design we will test our program’s impact, while rigorously evaluating our implementation framework. The designed intervention to be delivered in the ENJOY IMP-ACT aims to comprehensively tackle various aspects identified in our previous work that will support better uptake of park-based physical activity: ongoing supervised-induction sessions, structured volunteers training, and promotional activities including engagement with local community health centres. Furthermore, the economic evaluation of the social-return-on investment, and the extensive quantitative and qualitative evaluations, will provide new data to further enhance our understanding of the factors that can facilitate greater engagement of physical activity, and the cost associated with the creation of older-person designated active space. Knowledge of the investment and associated activities cost relative to the social return generated by the investment is likely to impact on future decision process within local governments around design, development and upgrades of outdoor recreational spaces. Hence, outcomes from this study have the potential to inform scale up across Australia with the goal of changing the trajectory of chronic disease and ill health of older people and can lead to a transformative shift in health policy of outdoor design.

## Data Availability

Not applicable.

## Abbreviations

ENJOY: Exercise interveNtion outdoor proJect in the cOmmunitY
SOPARC: System for Observing Play and Recreation in Communities
IMP-ACT: IMProving older people’s health through physical ACTivity
EQ-5D-5L: The 5-level EQ-5D
WHO-5: World Health Organisation (WHO-5) Wellbeing questionnaire
TREND: Transparent Reporting of Evaluations with Nonrandomised Designs
TERM: Training, Engagement, Resources development, Marketing and promotion
QR: Quick Response
